# Observations on Milk Sickness

**Published:** 1847-08

**Authors:** J. H. McNutt

**Affiliations:** Annapolis, Parke County, Indiana


ARTICLE III.

*Observations on Milk Sickness.* By Dr. J. H. McNutt, of Annapolis, Parke County, Indiana.

Much has been written in the form of communications to the various Medical Journals, respecting the disease known as *Milk Sickness* or *Trembles*, by our western practitioners; and some of the papers drawn up evince much careful investigation with regard to that formidable malady that may almost be classed with the opprobria medicinæ, and yet, notwithstanding the ability with which the subject has been handled, very little is known concerning the nature and pathology of this disease, that can be relied on, in a clinical point of view; but, like all other unsettled matters in pathology, each practioner has his own theory from which he draws deductions that, for a time, satisfies his own mind.

To review all the various and discrepant opinions that have been held respecting the cause of milk sickness, would extend this article beyond a practical length. I shall only notice some of them briefly.

I believe the first notice, in the form of a monograph, we have of milk sickness may be found in the Medical and Philosophical Lyceum, page 87, vol. 1st., by Dr. Drake. The symptoms of what he calls a new disease, are in part thus given: "It almost invariably commences with general weakness and lassitude; a strange propensity to sleep occurs. The skin is cool, pale, and frequently affected with clamminess, a disagreeable burning sensation in the stomach and hot evacuations—the thirst is urgent." The above is a very good description of this disease, at that period; and so far as treatment was then concerned it was mostly left in the hands of the nurses.

There have been three theories held in relation to the cause of milk sickness.

1st. That it is propagated by a vegetable poison eaten by our domestic animals, and introduced into the human system by eating the fresh milk or butter.

2d. That it is of atmospheric origin, and governed by the same laws that govern all malarial diseases.

Lastly, that it is a mineral poison, held in solution in the springs in certain localities.

Each of these theories has had its advocates, and I only notice the discrepancy of opinion with regard to the pathology of milk sickness, not for the purpose of entering the arena of controversy, but to stimulate all to the proper investigation. I will merely give my own views, aided by the lights that an experience of some years' practice in a milk sickness region has, from time to time, shed on the obscure subject, however different these views may be from those entertained by Dr. Wright as published in the Western Medical and Physical Journal, or to the mineral hypothesis of Seaton, Taylor, &c. I am indebted to that able pathologist,

Dr. Drake, for much valuable information as to the probable cause of milk sickness. The readers of the Western Medical and Surgical Journal are familiar with his views and as they are in accordance with my own, I more readily refer to them. I assume the ground in the premises that the disease is propagated by a vegetable poison. And here I might enter into a lengthy argumentation of hypothetical reasoning, but in doing so I would only be adding my mite to the many conjectures that run through our medical periodicals. If I can add anything that will be of advantage in a curative point of view, I shall feel satisfied with the effort.

I believe that milk sickness may, with a great deal of propriety, be divided into three stages, viz.: The initial, the febrile, or stage of excitement, and the congestive or stage of collapse, which are characterized by the following symptoms: In the premonitory or forming stage, there is not much disturbance in the circulation; it is nearly normal. There is rather a loathing of food; the thirst is not very great; the eyes show more clearly in this stage what is going on than any other pathognomic means we have. They have a dull heavy appearance as if covered by a pseudo-membranous substance. This stage generally lasts from 24 to 48 hours; and when the proper means are used, the disease may be frequently broken up in limina.

In the stage of excitement, the symptoms are more urgent than in the first stage. There is an increased action in the heart and arteries, with a tendency to coma. The eyes, in addition to the dull and heavy appearance above indicated, show an increased vascularity in all their capillary vessels. There is also a great deal of gastric irritability, with an almost indomitable disposition to retching and vomiting without being able to eject anything from the stomach, except a white glairy matter. There is a peculiar fetor about the breath that is usually developed at this time, which is one of the best diagnostic symptoms we have, and which, of itself, is sufficient to render the disease *sui generis*. This, to one of sensitive olfactories, who is accustomed to the disease, need never be mistaken. As is generally the case when there is gastric irritability there is disturbance in the action of the bowels. Here there is the most obstinate constipation, which is one of the principal difficulties in a therapeutic point of view. This period in the history of the disease is also characterized by extreme restlessness and jactitation, and what authors term a general feeling of malaise. The thirst is urgent, and the patient loudly calls for cold water which, for a few minutes, seems to be very grateful, and to allay the gastric distress and that intolerable burning sensation in the stomach; but even the water is retained but a short time until it is ejected from the stomach, and an urgent call for more, which is all the patient craves. This stage frequently lasts two or three days without any appreciable difference.

In the congestive or last stage, in addition to the symptoms already detailed, there is cold extremities; the skin has a dark livid and corrugated appearance. The superficial vessels seem collapsed as if entirely empty. The whole surface is covered with a cold clammy sweat. This stage cannot last long until a crisis takes place, either in convalescence, or the patient becoming moribund.

Having thus briefly glanced at the most prominent symptoms characteristic of the disease, I will close by giving what I consider the most efficient treatment; and first the following indications seem naturally to present themselves, viz.: to allay the gastric irritability, and thereby remove obstructions from the bowels; and secondly, restore the balance in the circulation.

The question comes up, what are the means that will best fill these indications? I might here remark that milk sickness is frequently complicated with other diseases. I believe it is a law that governs all diseases, that epidemics have an influence on such diseases as are propagated by local specific causes. Such amalgamation more frequently supervenes when we have milk sickness during the presence of our autumnal fevers. The same order will be observed in giviug the treatment that was had in delivering the symptoms.

In the initial or forming stage, strict dietetic rules should be observed, and the bowels be kept in a soluble condition. Active physical exertion is known by all conversant with the disease to have a prejudicial effect. Hence it is that cattle bought in a milk sickness region, although apparently healthy when collected, it is no uncommon thing for a number of them to take the trembles on being driven the first few miles.

The next is the stage of excitement; and as the lancet has, for a long time, been regarded as one of the most important curative means in all febrile diseases, the propriety of its use will be briefly considered *a nulla discrimina.*

Depletion with the lancet, in febrile diseases, has obtained and held the empire in medicine too long. Without stopping to inquire what is the grade and variety of fever we are treating, and what are the indications we expect to fill by this sanguinary treatment, I am not of that school who believe it to be reprehensible in all diseases; but we should never bleed where there is fever, guided alone by the simple fact that we have done so before, in some variety of fever, and that, too, with advantage to our patient. Here we have one variety of fever, and that, if not primarily congestive, with at least a strong congestive tendency throughout its course. Now the question comes up, what are the therapeutic means that will best increase the centrifugal action of the heart and arteries? All will give the same response, whether guided by experience or sound pathology. More might be said on the point of the treatment; but since pathology has shed its benign influence on our science, empiricism has fallen before its rational inductions.

Emetics are sometimes highly useful means, especially in that variety that is complicated with our autumnal fevers. Here the treatment might be premised by a mild emetic of pulv. ipecac. to unload the prima via. Having done this, let us dispense with the further use of this class of agents, and the decided preference is given to ipecac. as having less tendency to produce prostration than most other articles of this class.

Cathartics stand at the head of our remedial means; and, as in many other diseases, a proper choice of the articles we use is of the utmost importance. When called to an ordinary case of this disease, if there is much gastric irritability, the first thing is to allay that; and for the purpose twenty or thirty gt. tinc. opii. given in the effervescing draught, will have a happy effect; or one fourth gr. of the acetate morphia, after which give twenty-five grs. sub-murias hyd. If it should not move the bowels in three or four hours, give at least two ounces ol. ricini, in conjunction with twenty or thirty drops of spirits of turpentine; and if that should be either rejected by the stomach or fail to operate, repeat it at intervals of three or four hours until the bowels are freely moved, assisting by suitable enemata. Much may be done at this stage by perseverance. Let all who have charge of cases of this character reflect that there is here no time for temporising. After having freely evacuated the bowels, the next thing is to keep up the alvine ejections; and this is best done by the frequent administration of castor oil and spirits turpentine, as above indicated.

At this period, the disease frequently passes into the third stage: and here tonics or stimulants are imperiously demanded, and I believe no class of tonics will answer a more valuable purpose than the sulphate of quinia. There is more analogy between milk sickness at this stage and congestive fever than any other disease; so much so, indeed, that some very respectable members of the profession have denied the existence of milk sickness altogether, and treated all such cases as congestive fever.

I cannot better illustrate the happy effects of the sulphate of quinine than by giving the history of the following case:

Mrs. G., aged 30, of strong constitution, was taken rather violently with the milk sickness, accompanied with all the usual symptoms. Was seen by me on the third day after attack, at which time the disease had passed into the second stage. There were some cerebral disturbances; constipation of the bowels, and constant efforts to vomit. Thirst great with considerable general arterial excitement. A large blister was laid over the epigastric region. She was ordered the following: sub-mur. hyd., twenty grains, and to be followed by oleum ricini and spirit. terebinthina. Next day visited her. Her bowels had acted slightly. All the symptoms greatly aggravated. Almost furious delirium. Extremities cold. Patient in the third or congestive stage of the disease. And here I would remark that I regarded the cephalic derangement as more dependent on congestion of the brain than on inflammatory condition; and, to use a common expression, I must acknowledge that I felt rather stumped. My patient was evidently sinking. Tonics or stimulants must be used—their use contra-indicated in consequence of the cerebral derangement. I had often thought of the quinine as being a good equalizer, and as admirably calculated to overcome the congestion of the heart and large vessels and here was the case. I ordered the following:

℞.—Pil. Hyd., grs. xii;
Quinia, grs. xxiv;
Mic.—Fiat Pilulæ viii.

One of these to be given every hour until all were taken. These were given through the night; and in the morning I had the gratification of finding my patient improved. Her bowels had been freely moved, bringing away dark bilious evacuations. I ordered mucilaginous drinks through the day, and at night the following, viz.:

℞.—Pil. Hyd. grs. viii;
Sulph. Quinia, grs. xviii;
Mic.—Fiat Pilulæ vi.

Of these, one every two hours, and to be moved off with the castor oil and spirits of turpentine. This treatment was continued until the balance in the circulation was restored; after which the patient was ordered to keep her bowels open with castor oil and turpentine, and a light farinaceous diet.

I have since treated other cases in the same manner, and find the quinine a most valuable adjuvant in filling one indication in the treatment of the disease, viz.: restoring the balance in the circulation, and overcoming congestion.

Blisters are perhaps next in importance in the treatment of this disease. Where there is much disposition to vomit (which is generally the case), their early use is clearly indicated. A large blister should at once be laid over the region of the stomach; and should the extremities become cold, blisters to the ancles and sinapisms to the wrists are indispensable. Enemata are highly useful means in overcoming the torpor of the bowels which is characteristic of the disease. The bowels are frequently costive from the fœces becoming indurated in the rectum; which may be overcome by the solvent effects of injections. There are other articles that may be used with advantage in the treatment of the disease. The effervescing draught is very grateful, but, as was before remarked, our reliance is to be placed on a few remedies. Such is a brief history of the disease, with what I consider the best treatment hitherto pursued.

